# Neuroplasticity-Based Cognitive and Linguistic Skills Training Improves Reading and Writing Skills in College Students

**DOI:** 10.3389/fpsyg.2013.00137

**Published:** 2013-03-25

**Authors:** Beth A. Rogowsky, Pericles Papamichalis, Laura Villa, Sabine Heim, Paula Tallal

**Affiliations:** ^1^Center for Molecular and Behavioral Neuroscience, Rutgers UniversityNewark, NJ, USA

**Keywords:** language, writing, reading, neuroplasticity, computer-based instruction, cognitive skills training

## Abstract

This study reports an evaluation of the effect of computer-based cognitive and linguistic training on college students’ reading and writing skills. The computer-based training included a series of increasingly challenging software programs that were designed to strengthen students’ foundational cognitive skills (memory, attention span, processing speed, and sequencing) in the context of listening and higher level reading tasks. Twenty-five college students (12 native English language; 13 English Second Language), who demonstrated poor writing skills, participated in the training group. The training group received daily training during the spring semester (11 weeks) with the Fast ForWord Literacy (FFW-L) and upper levels of the Fast ForWord Reading series (Levels 3–5). The comparison group (*n* = 28) selected from the general college population did not receive training. Both the training and comparison groups attended the same university. All students took the Gates MacGinitie Reading Test (GMRT) and the Oral and Written Language Scales (OWLS) Written Expression Scale at the beginning (Time 1) and end (Time 2) of the spring college semester. Results from this study showed that the training group made a statistically greater improvement from Time 1 to Time 2 in both their reading skills and their writing skills than the comparison group. The group who received training began with statistically lower writing skills before training, but exceeded the writing skills of the comparison group after training.

## Introduction

While reading instruction is the focus of early literacy skills, as students move into the high school and college years there is increasing focus on writing. By the time U.S. students reach high school it is assumed that they have already learned to spell words and use punctuation in standard ways and that the words and syntax they use in their writing comply with the rules of Standard Edited American English (SEAE) grammar (National Commission on Writing, 2003). Despite the importance for students to become proficient in SEAE writing skills, the National Assessment of Educational Progress’ (NAEP) most recent writing assessment showed that only 27% of U.S. students in grade 12 performed at or above the proficient level (National Center for Education Statistics, 2012). Because of the increasing importance of writing for success in college, assessment of proficiency in written SEAE now comprises one-third of the Scholastic Aptitude Test (SAT^®^; College Board, 2012a). Nearly all 4-year college and universities in the U.S. (including test-optional institutions) use students’ SAT scores as a measure of college readiness as well as an indicator of likely college success from students of all backgrounds. The SAT provides subject-level readiness indicators for both Critical Reading and Writing measures. The college enrollment rate of 2011 U.S. high school graduates was 72.35% for young women and 64.6% for young men (U.S. Department of Labor, Bureau of Labor Statistics, 2012). With such a high number of students enrolled in college one would assume reading and writing scores to be high for this population of students, as college places such heavy demands on both reading and writing. Unfortunately, less than half of 2012 college bound seniors met the SAT College and Career Readiness Benchmark for Critical Reading and Writing: 49% of students met the critical reading benchmark, 51% did not; and 45% of students met the writing benchmark, 55% did not (College Board, 2012b). As the majority of 12th grade students continue to fail to reach proficiency in writing skills many colleges are increasingly faced with providing developmental writing instruction to their students. This may be especially important to those colleges that have a high proportion of students with English as a second language (ESL) and underrepresented minority students.

These data clearly point to the need for more research on the efficacy of writing instruction and intervention strategies for college students who are continuing to struggle with writing. Specifically, there is a need for a better understanding of the foundational cognitive, linguistic, and reading skills important for proficient writing as well as the development of scientifically validated methods and intervention strategies for improving writing at the college level. At the college level, it is also important to develop methods that can be standardized, scaled with efficiency for individualized use, and demonstrated to generalize broadly to writing in a variety of contexts. Recently, there has been a growing focus on the development of neurocognitive approaches; particularly those based on neuroplasticity research, for improving language and literacy skills (Kujala et al., 2001; Habib et al., 2002; Song et al., 2012). The purpose of the present study was to assess the impact of computer-based cognitive, language, and literacy skills training on the reading and writing skills of college students with demonstrated writing deficiencies. In this study, we address whether intervention strategies derived from physiological and cognitive neuroplasticity research may provide a novel approach for addressing the needs of twenty-first century college students who continue to struggle with writing.

### The language to literacy continuum

It is our premise that if we want to create more effective instructional and intervention methods for assisting struggling learners, it is important to better understand the causes and determinants of individual differences in the development of higher cognitive skills such as writing. Put simply, we need a better understanding of the foundational neurocognitive, and linguistic skills on which proficient writing depends from a developmental perspective. Graham and Perin (2007) explain that reading and writing are complementary skills that run a roughly parallel course with language. Writing is putting words on paper. Words come from what students hear, speak, and read.

Spoken language is the foundation of written language. In order to break the code for proficient reading, which is linked to proficiency in writing, students must become phonemically aware that words can be broken down into smaller units of sound (phonemes) and that it is these sounds that the letters represent (Lyon, 1995; Castles and Coltheart, 2004). In their earliest reading and writing experiences, students are instructed to listen for the relationships of sounds to letters, which contributes to their phonemic awareness and knowledge of phonics. In addition to the large body of research showing that explicit training in phonemic awareness improves reading (see National Reading Panel, 2000, for review), explicit phonemic awareness training also has been shown to improve writing both in typically developing and at risk students. In a study with typically developing students, Eldredge and Baird (1996) found that students increased their ability to write when a structured phonics program was used as compared to a holistic or whole language approach. The analysis of student’s writing revealed that those who received a structured phonics program as compared to those receiving holistic instruction wrote using more words (*p* < 0.002), different words (*p* < 0.002), difficult words (*p* < 0.03), and composition units (*p* < 0.002). In addition, the students in the structured phonics group also surpassed those in the holistic group on the number of words spelled correctly (*p* < 0.026) in written compositions. Finally, the study showed that the overall quality of the written compositions by the structured phonics group were significantly higher than the students in the holistic group with an effect size equivalent to a 49 percentile point difference in performance on this variable. While studies such as this one have typically focused on beginning writers, it is important to note that studies with struggling readers have shown that phonemic deficits continue to occur even in college students with a history of reading impairments (Gallagher et al., 1996; Wilson and Lesaux, 2001; Cirino et al., 2005; Callens et al., 2012).

In addition to phonemic awareness, reading and writing also depend on other foundational language abilities including semantics, morphology, and syntax (Byrne, 1981; Joanisse et al., 2000; Catts et al., 2002; Carroll and Snowling, 2004). As students progress in reading into the middle school years, morphological awareness plays an increasingly important role in literacy development (Singson et al., 2000). There is a systematic progression of grammatical morpheme acquisition which includes present progressives (-ing), plurals, irregular past forms, possessives, articles, regular past tense verbs, third person singular, simple present tense, and *be* verbs (Lightbown and Spada, 2002). Not only is literacy success dependent on adequate acquisition of these morphological structures, but also on a student’s correct application of syntactical rules, for example, in English, the proper temporal order of words within phrases and larger units–e.g., adjectives before nouns. The connection between speaking, reading, and writing is an ongoing cycle with speaking, reading, and writing supporting one another. As they write, most individuals mimic the oral language they hear internally (Carrow-Woolfolk, 1996). While oral expression generally develops without the need for explicit instruction, written communication requires much more deliberate effort and intensive practice to learn to communicate ideas effectively and accurately. When listening or reading, the student passively experiences language structures. Writing, on the other hand, requires students to actively focus on language structures and written conventions in order to learn to reproduce them in written form.

### Perceptual and cognitive prerequisites for literacy

There is a large body of research demonstrating a link between individual differences in foundational perceptual and cognitive skills and individual differences in language and reading development and disorders (Farmer and Klein, 1995; Kraus et al., 1996; Stein and Talcott, 1999; Habib, 2000; Wright et al., 2000; Tallal, 2004). Similarly, foundational cognitive skills (memory, attention, processing speed, and sequencing) have also been posited to underlie individual differences in writing. Berninger and Winn (2006) proposed the “not-so-simple-view” of writing. This model asserts that an individual’s level of writing competence relies on the efficiency of the writer’s cognitive abilities. Multiple areas of the brain must work together to produce writing that conforms to the rules of SEAE. Sentence generation involves consciously reflecting on and manipulating knowledge which needs to be retrieved rapidly from long-term memory or actively maintaining it in short-term working memory with some level of automaticity and with disregard to irrelevant information. Writing consumes the writer’s full attention as the writer thinks about what to say and applies correct spelling and syntactical rules to what is written. As the writer fixates on each word in a sentence, all preceding words in that sentence must be maintained in working memory while simultaneously selecting new words in their correct sequence to form correct sentences and paragraphs that convey the intended thoughts.

To write using SEAE, students must remember what they want to write, pay attention to the way they write it (correct spelling, capitalization, and punctuation), and construct what they want to write so that it appears on paper in a logical sequential order using correct vocabulary, morphology, and syntax. Understanding the developmental progression of language skills, coupled with the increasing role of foundational cognitive skills as students attempt more advanced writing seems particularly important in the design of instructional methods to improve writing. Given the strong relationship between basic perceptual, cognitive, spoken, and written language skills, coupled with evidence that early patterns of deficits in struggling students continue to be evident (but are rarely addressed) in older students, we hypothesized that structured methods that explicitly focus on improving basic perceptual and cognitive skills in the context of increasingly challenging language and reading comprehension would result in improvements in reading and writing skills in college students who continue to struggle with writing.

### Using neuroplasticity-based training programs to enhance foundational perceptual, cognitive, and linguistic skills

Tallal et al. (1996) and Merzenich et al. (1996) were the first to develop training programs for students with language-based learning deficits that explicitly focused on improving underlying perceptual and cognitive skills in the context of language. Their methods were based on neuroplasticity research in animals that showed that the functional organization of the brain at the cellular level could be changed and behaviors improved by intensive behavioral training (Recanzone et al., 1993). Several training principles were found in these animal studies to be necessary for driving neuroplastic changes in the brain, both at the physiological and behavioral level. These include intensity and frequency of trials, focused attention to a task, individually adaptive (easy to hard) trials, and timely rewards and correction of errors to reinforce learning and maintain motivation.

In order to evaluate whether neuroplasticity-based training could be used to improve language skills in children, Tallal et al. (1996) and Merzenich et al. (1996) developed a series of neuroplasticity-based listening training exercises disguised as computer video games. The exercises were broadly designed to drive neuroplastic changes in attention, processing speed, sequencing, and memory within the context of training language skills from the phonological to the grammatical level. In their first study, they evaluated the efficacy of this approach with children with specific language learning impairments (LLI). Children were quasi-randomly assigned to two matched groups. The language impaired children in both the experimental and control group received the same intensive speech and language intervention over a 4-week training period. However, the experimental group received the training with speech that had been acoustically modified to increase the amplitude and duration of the fastest changing (3–30 Hz) components within syllables and words, while the control group received the training with regular (not modified) speech. In addition, the experimental group played a video game that was designed to individually adapt to increase their rate of auditory processing, while the control group played a visual video game for the same period of time that did not vary the rate of stimulus presentation. Results showed that neuroplasticity-based training could significantly improve basic auditory processing speed thresholds (Merzenich et al., 1996). Furthermore, while both groups improved in language abilities after the intensive listening training program, the improvements in language abilities were significantly greater for the experimental group (Tallal et.al, 1996). This series of linguistic exercises and video games formed the basis for the Fast ForWord^®^ series of language and reading programs (www.scientificlearning.com).

There are several studies with mixed results that have focused on the effectiveness of the original Fast ForWord Language product for children with specific language impairment or dyslexia (Temple et al., 2003; Troia and Whitney, 2003; Cohen et al., 2005; Gaab et al., 2007; Gillam et al., 2008; Given et al., 2008; Stevens et al., 2008) or rehabilitation of cognitive skills in elderly adults (Szelag and Skolimowska, 2012). The NIH-randomized control trial is the most recent and comprehensive of these studies (Gillam et al., 2008). In this study, 216 children between the ages of 6 and 9 years with language impairments were randomly assigned to one of four conditions: (a) Fast ForWord Language, (b) academic enrichment, (c) computer-assisted language intervention, or (d) individualized language intervention provided by a speech-language pathologist (SLP). All children received 1 h and 40 min of treatment, 5 days per week for 6 weeks. Language and auditory processing measures were administered to the children by blinded examiners before treatment, immediately after treatment, 3 months after treatment, and 6 months after treatment. Gillam et al. (2008) found that children who interacted with computers during their intervention time using Fast ForWord-Language or computer-assisted language intervention (CALI), fared as well as children who received one-to-one individual language intervention with a certified SLP or academic enrichment, all of whom made highly significant improvements on standardized language measures. Furthermore, at the immediate post-test, as well as the 3- and 6-month follow-up testing, participants who were trained with the two computerized instructional programs (Fast ForWord-Language or CALI) that focused primarily on auditory discrimination of sounds, syllables, and words, yielded better phonological awareness results than the computerized treatment that focused on general academic skills or the clinician-directed language treatment. Gillam et al. (2008) concluded that all conditions examined in this study yielded highly significant improvements in language, indicating that intensity of intervention may be the driving factor across conditions. However, they also pointed out that when comparing interventions one should consider that the cost and time investment of the SLP is greater than that of the computerized interventions that were delivered to groups of children.

All of the previously published studies have evaluated the efficacy of the original Fast ForWord-Language products in younger students. Over the years, a much broader series of more advanced exercises have been developed for middle and high school students. Fast ForWord Literacy (FFW-L) and Fast ForWord Reading (FFW-R Levels 3–5) software engage more advanced students in a series of listening, language, and reading exercises designed to build higher-level cognitive and language-based literacy skills. As students progress through the exercises of FFW-L and the FFW-R series, the demands on working memory, attention span, processing speed, and sequencing are continually increased within the context of increasingly complex linguistic material. To our knowledge, no previously published study has focused on college students or assessed the efficacy of a combination of the FFW-L and higher-level Reading (Levels 3–5) products in improving reading and writing. The present study explored the following main research question: does the Fast ForWord program, aimed at improving basic through advanced cognitive, language, and reading skills, impact college students’ reading and/or writing skills? Specifically, this study evaluated the effectiveness of the FFW-L and FFW-Reading exercises in improving reading and SEAE writing skills in college students with poor writing skills.

## Materials and Methods

### Research design

This study used a quasi-experimental research design with two groups; below average and good writers. The intent of the experiment was to determine whether providing intensive cognitive, language, and reading training to college students with below average writing skills would generalize to improved writing abilities. In a quasi-experimental research design subjects are not necessarily equal on variables of interest (in this study writing and reading skills) and are not randomized across treatment and control groups.

### Participants

The participants in this study were 53 college students enrolled in an urban public university located in northern New Jersey. Approximately 6,000 undergraduate and 4,000 graduate students attend the university. The university has maintained a longstanding commitment to recruiting and supporting ethnic minorities from the surrounding community. Two populations of undergraduate students with historically lower literacy abilities were invited to participate in this study’s training group. The first population consisted of students enrolled in a developmental writing course during the 2010 fall semester. If students do not meet the required competencies for enrolling in Composition 101, they are required to pass non-credit developmental writing coursework before they are allowed to enroll in the required Composition classes. The second population of students invited to participate in the training group consisted of students enrolled in the Louis Stokes Alliance for Minority Participation (LSAMP) program. The LSAMP program is aimed at increasing the quality and quantity of underrepresented minority students successfully completing a science, technology, engineering, or mathematics (STEM) baccalaureate degree. Students from the general population of college students at the same University were recruited to participate in the comparison group. All of the students who volunteered were accepted into the study and signed a formal letter of consent. This study was reviewed and approved by the university’s Institutional Review Board.

The training group included 25 students, 17 females and 8 males with a mean age of 20.08 years (±3.57). The comparison group included 28 students, 16 females and 12 males with a mean age of 19.39 years (±1.37). Gender ratio was similar across groups (χ^2^ = 0.66, df = 1, *p* < 0.416). ESL distribution was significantly different between the two groups (χ^2^ = 5.37, df = 1, *p* < 0.021); the training group consisted of 13 ESL and 12 non-ESL students while the comparison group had 6 ESL and 22 non-ESL students.

### Computer-based cognitive and literacy skills training tasks

The computer-based cognitive and literacy skills training used in this study was FFW-L (Scientific Learning Corporation, 2006) followed by Fast ForWord Reading, Levels 3–5 (FFW-R3, R4, R5; Scientific Learning Corporation, 2011). The training was designed specifically for secondary students with a focus on increasingly demanding cognitive, listening, and reading skills. The exercises individually adapted to increasingly challenge student’s memory, attention, processing speed, and sequencing within the context of increasingly demanding spoken and written stimuli. The training provided students with (a) an orienting button that allowed the student to control when each trial was presented (b) frequent stimuli that required sustained attention and a response on each trial, (c) trials that adapted to each student’s responses, mouse-click-by-mouse-click, moving from easy to harder trials, and (d) timely feedback, correction of errors, and rewards after each correct response. Exercises in both the literacy and reading series trainings made use of modeled grammatically correct language, repetition of content, instant feedback, individualized instruction, combined auditory and visual stimulation, and concentrated and continuous practice to enhance and automatize listening and literacy skills, all of which are critical components for improving writing. The overarching goal of the series of exercises was to progressively drive more efficient and consistent neural processing as well as to improve performance in the linguistic domains of phonology, semantics, morphology, and syntax within both spoken and written English. However, no explicit practice with writing *per se* was included in the training. Example screen shots and a brief explanation of each of the training exercises used in this study are provided in Supplementary Material.

#### Language/listening training

Students began with a language/listening training program (FFW-L) that included a series of six training exercises each designed to build auditory perceptual, cognitive, and linguistic skills important for spoken language comprehension. Exercises in this program aimed to help students increase their focused attention and working memory span for auditory/spoken information and strengthen listening comprehension by having students work with auditory sequences, spoken phonemes, morphemes, words, sentences, paragraphs, and full stories. Exercises focused on the use of grammar in sentence context and systematically trained all of the rules of English grammar.

#### Reading training

Once students completed the language/listening program they progressed to the reading training programs (FFW-R3, R4, & R5). These exercises used a similar format and specifically built upon the cognitive and language skills developed in the first program, but in this case with a focus on written language. These exercises progressed at each student’s own pace from a simple focus on letter-sound correspondences, to building an understanding of grammatical morphology, training in writing conventions (i.e., spelling, punctuation, and capitalization), and sentence and paragraph construction. As students progressed, the exercises required increasing use of executive functions within the context of increasingly challenging linguistic contexts.

#### Implementation of training

Students in the training group trained 50 min per day, 4–5 days per week for 11 weeks in a computer lab on campus. Students used the software series for 32–50 days (*M* = 42; SD = 5.4). Each training exercise had a set amount of content to complete, and each student completed this content at his/her own pace depending on the number of trials attempted and errors made. As such, each student completed different amounts of content. All 25 students in the training group completed the language/listening series, and advanced to varying levels of the reading series. Attendance, which is the number of days attended relative to the weekdays available during the training period, was 84% (SD = 0.11). Participation, indicating the time on task on the days students were present, was 103% (SD = 0.04), indicating that the average student was sufficiently engaged and motivated by the software to participate for slightly over the required 50 min per day.

A certified English teacher and research assistants who were responsible for assuring compliance with the software’s training protocol monitored the training. The teacher completed the training provided by the developers of the software in the use of this software, as well as how to interpret students’ daily results in order to provide help for students who were struggling with specific aspects of the program. Students were introduced to the program on the first day of training and practiced each exercise using the demonstration examples provided with the software to assure they understood each exercise. Each day thereafter, students accessed a computer in the lab and entered their password to start the program. The passwords assured that each student’s data were retained and uploaded to the software’s progress monitoring tools after each session. As a result, the program started each subsequent session where the student stopped the previous session.

Each exercise is individually adaptive, moving to harder items based on correct responses and back to easier items based on errors. The exercise progression algorithm aimed to keep each student performing at approximately 80% correct and students had to master easier content items before moving to more difficult items. Thus, the percent of content completed in each exercise, rather than percent correct, was the data of interest. The software’s progress monitoring tools kept daily records of each student’s percentage of completion on each exercise and prompted the teacher as to when the student had completed the language/listening program and was ready to begin the reading series. The teacher also received real-time feedback in the form of daily progress graphs and detailed reports of errors that indicated need for intervention. The software’s online data management system provided red flags to the teacher that pinpointed individual students who were struggling with specific exercises. As part of the accepted best practices in the use of this software, the teacher and research assistants were trained how to use this online progress monitoring system and encouraged to interact with students based on this feedback. The daily progress reports gave the teacher explicit suggested interventions she could use to help the student progress through the content in each exercise. While the teacher was assisting an individual student, research assistants circulated throughout the lab monitoring student progress.

### Assessment measures

#### Gates MacGinitie reading test

Students’ reading comprehension was measured by the fourth edition of the online version of the comprehension subtest of the Gates MacGinitie Reading Test (GMRT) (MacGinitie et al., 2010). The GMRT is a timed, group-administered assessment of reading comprehension. The Adult Reading (AR) level of the test can be administered on more than one occasion, alternating between forms S and T. Alternate form reliability is reported as 0.83. The AR comprehension section consisted of 11 expository and narrative passages, each followed by three to six multiple-choice questions, for a total of 48 questions. Students read each passage silently and then answered three to six multiple-choice questions related to the most recently read passage. Items increased in difficulty as the student progressed through the test during the 35-min time limit. Internal consistency reliability is reported as 0.85.

#### Oral and written language scales written expression scale

Students’ writing was measured by the OWLS Written Expression Scale (Carrow-Woolfolk, 1996). The OWLS Written Expression Scale is a standardized assessment of written language skills that can be administered individually or in small groups to individuals 5 through 21 years of age. The OWLS Written Expression Scale was chosen because of its high reliability and relevancy to authentic writing. The *OWLS Manual: Written Expression Scale* reports a test-retest evaluation with 9 weeks between administrations and found no improvement on the students’ scores at any age. The internal consistency of the OWLS Written Expression Scale was 0.88; test-retest reliability was 0.88; and inter-rater reliability was 0.95. In addition to its high reliability, the OWLS Written Expression Scale addresses the elements of writing commonly assessed in standardized high stakes tests. These elements include use of content (meaningful content, details, relevance, coherence, supporting ideas, word choice, and unity), linguistics (modifiers, phrases, question forms, verb forms, and complex sentence structure), and conventions (spelling, letter formation, punctuation/capitalization, and conventional structures). Items on the assessment consisted of both structured and open-ended writing tasks that represent typical writing activities found in the classroom, thus providing a broad and extensive sample of an individual’s writing skills. For example, in one question, students are asked to write a paragraph describing why they prefer cats or dogs. Another question asks students to describe a bicycle to an alien using a single, well formed sentence. Yet another question provides data in a table for students to interpret and asks the students to write a paragraph describing these data.

### Data analysis

In the current study, the GMRT and OWLS Written Expression Assessment were administered to all participants, both before (Time 1) and after (Time 2) the training group received the training. Different versions of the same test were available for the GMRT, but not for the OWLS. The GMRT was scored automatically by a computer automated scoring program provided by the test developer. Computer automated scoring of the OWLS Written Expression Assessment was not available. Rather, the OWLS requires a trained professional experienced in scoring this test using the standardized rules and examples explained in the *OWLS Manual* to score each item. To increase reliability and reduce potential bias in this study, the training group and comparison group’s tests were intermixed so that the scorer was blind to whether they were scoring a training or comparison group subject. To increase consistency in scoring, individual student’s Time 1 and Time 2 tests were scored together. However, the order of scoring Time 1 and Time 2 tests was randomized so that the scorer did not know which test was being scored at any time, Time 1 or Time 2. To further ensure consistency, the tests were scored methodically, scoring all of question 25 for all subjects, and then all of question 26, and so forth. Thus, during the scoring process the scorer was blind to whether they were scoring a response from a trained or comparison subject or from Time 1 or Time 2. As per instructions in the *OWLS Manual*, raw scores were converted to grade-based norm standard scores (*M* = 100, SD = 15).

A total of 106 Time 1 and Time 2 tests were scored for 53 subjects (25 training; 28 control). Two scorers experienced in scoring the OWLS Written Expression Scales participated in scoring the data for this study. Scorer A scored all tests. A second scorer (Scorer B) scored a selection of a total of 48 tests (the Time 1 and Time 2 tests of 24 study participants; 12 randomly selected from the training group and 12 from the comparison group). A Pearson correlation coefficient was calculated for the relationship between the two raters’ scores. A strong positive correlation was found [*r* (46) = 0.71, *p* < 0.01]. Next, all discrepancies were discussed between Scorer A and Scorer B, with reference back to the *OWLS Manual*, and a True score was determined. The vast majority of discrepancies centered on a small number of items. Each of those items were discussed and resolved between Scorer A and B and then those items were rescored for all subjects. The final correlation coefficient between Scorers A and the True score was [*r* (46) = 0.96, *p* < 0.01].

To examine the extent to which training affects changes in literacy measures, standard scores of the GMRT, and OWLS were first submitted to separate 2 × 2 mixed-model analyses of variance (ANOVAs), with the between-subjects factor being Group (training vs. comparison) and the within-subjects factor being Time (1 vs. 2). In a second step, we explored the effects of ESL on GMRT and OWLS outcomes by conducting 2 (ESL; no vs. yes) × 2 (Time; 1 vs. 2) mixed-factor ANOVAs. Because of the small number of ESL students in the comparison group, these analyses were restricted to the members of the training group. Partial eta-squared ($\eta_{p}^{2}$) values were reported as a measure of effect size (Cohen, 1988). Where appropriate, contrast analyses were used to follow-up significant ANOVA results. For all statistics, effects were deemed significant when *p* < 0.05.

## Results

**Figure 1:**
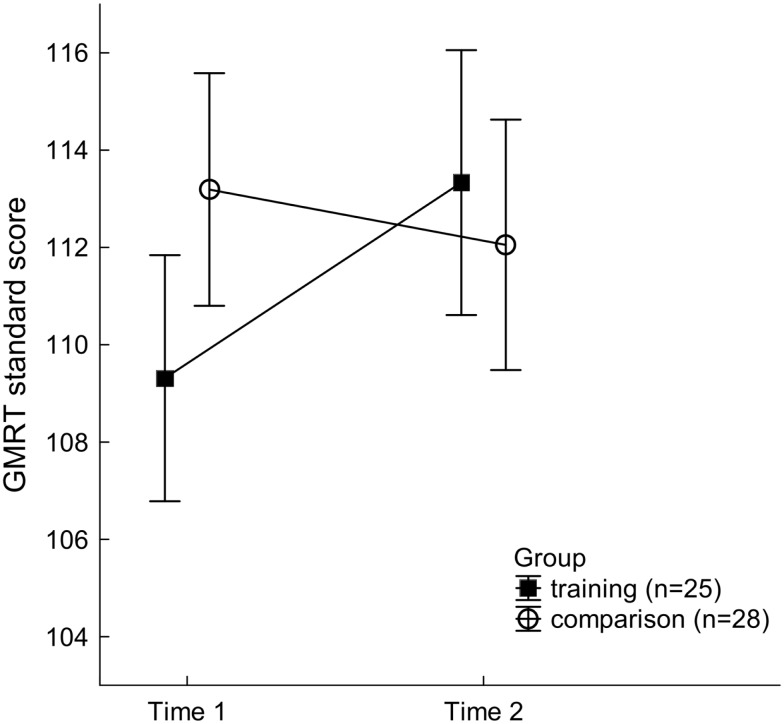
**Gates MacGinitie Reading Test (GMRT) standard scores for the two participant groups at Time 1 and Time 2**. Mean values of 25 training group students (filled squares) and 28 comparison students (open circles) are depicted. Vertical bars represent standard errors of mean. While there was no significant change in reading performance in the comparison group, GMRT scores in the training group increased significantly from Time 1 to Time 2.

Demographic characteristics and standardized literacy scores for the training group and comparison group are shown in Table 1. Students in the training group demonstrated systematic gains in both reading and writing skills following training. For the GMRT reading assessment, the two-way ANOVA revealed that main effects of factors Group and Time did not reach statistical significance. However, there was a significant Group × Time interaction, *F*(1, 51) = 4.31, *p* < 0.043, $\eta_{p}^{2}=0.08$ (see Figure 1). As evaluated by focused contrasts, this interaction was accounted for by significantly better reading scores at Time 2, post-FFW training, compared to Time 1, pre-FFW training, in the training group only, *F*(1, 51) = 4.96, *p* < 0.031.

**Table 1:** **Demographic characteristics and standardized literacy measures by participant group**.

	Training group	Comparison group
Sample size	*n* = 25	*n* = 28
Gender (male/female)	8/17	12/16
ESL (no/yes)	12/13	22/6
Age (years)	20.08 ± 3.57	19.39 ± 1.37
GMRT time 1	109.31 ± 11.77	113.19 ± 13.38
GMRT time 2	113.33 ± 13.03	112.05 ± 14.12
GMRT difference time 1 vs. 2	*p* < 0.031	*p* < 0.508
OWLS time 1	86.20 ± 9.68	98.11 ± 14.84
OWLS time 2	111.04 ± 15.88	95.61 ± 17.40
OWLS difference time 1 vs. 2	*p* < 0.001	*p* < 0.326

*Means ± standard deviations are shown; *p*-values are based on contrast analyses with a significance level set to 5%. ESL, English as a second language; GMRT, Gates MacGinitie Reading Test; OWLS, oral and written language scales, written expression scale*.

**Figure 2:**
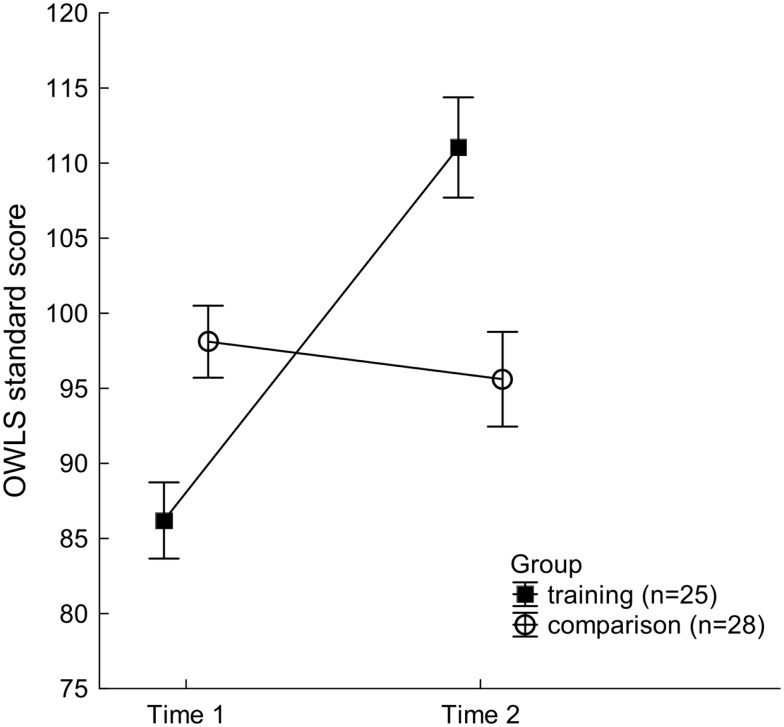
**Written Expression Scale standard scores of the Oral and Written Language Scales (OWLS) for the two participant groups at Time 1 and Time 2**. Mean values of 25 training group participants (filled squares) and 28 comparison participants (open circles) are shown. Vertical bars indicate standard errors of mean. While the training group students were outperformed by the comparison group at Time 1, their considerable spurt in writing following intervention, led to a reversed performance pattern at Time 2, with lower standard scores in the non-trained students.

For the OWLS Written Expression Scale, a significant main effect of Time was found, *F*(1, 51) = 37.14, *p* < 0.001, $\eta_{p}^{2}=0.42$, demonstrating that writing scores increased from Time 1 (mean ± SEM; 92.15 ± 1.74) to Time 2 (103.32 ± 2.30) across both groups. This was, however, mainly driven by the performance changes of the training group participants as evinced by a significant Group × Time interaction, *F*(1, 51) = 55.63, *p* < 0.001, $\eta_{p}^{2}=0.52$ (see Figure 2): follow-up contrasts indicated that the training group participants achieved significantly higher writing scores at Time 2, upon completion of FFW training, than they had at Time 1, before FFW training, *F*(1, 51) = 86.93, *p* < 0.001. No comparable improvement was found for the comparison group. Further, at the onset of the study (Time 1) the writing scores of the training participants were significantly lower than those of the comparison group, *F*(1, 51) = 11.65, *p* < 0.002. However, at Time 2, after the training group had completed the FFW training programs, their considerable improvement in writing led to a reversed performance pattern, with the trained group’s standard scores on the OWLS now significantly exceeding those of the comparison group, *F*(1, 51) = 11.28, *p* < 0.002.

**Figure 3:**
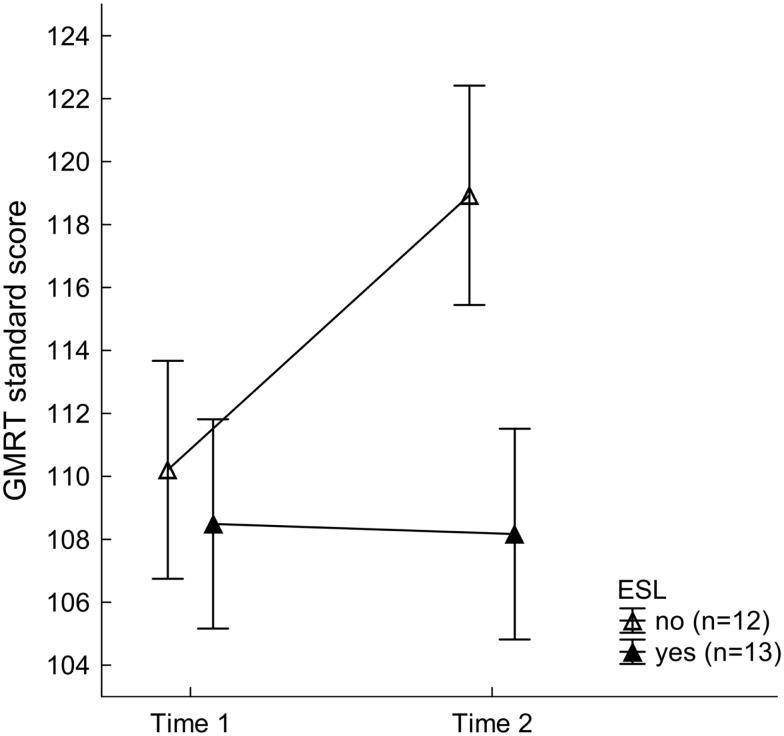
**Gates MacGinitie Reading Test (GMRT) standard scores for the training group as a function of speaking English as a second language (ESL) at Time 1 and Time 2**. Mean values of 12 non-ESL (open triangles) and 13 ESL students (filled triangles) are shown. Vertical bars represent standard errors of mean. Non-ESL training participants improved significantly across time and outperformed the ESL students after completion of the intervention protocol.

**Figure 4:**
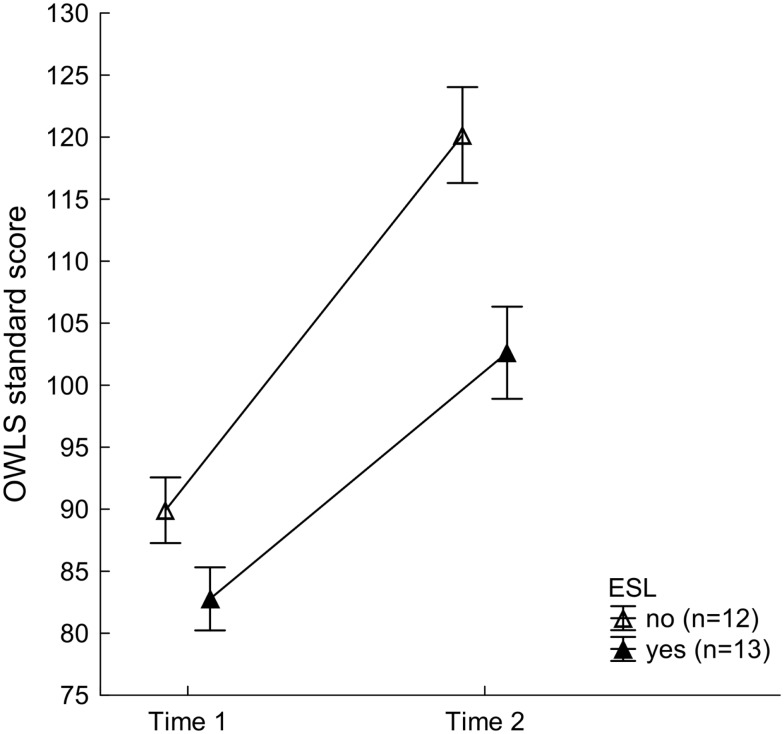
**Written Expression Scale standard scores of the Oral and Written Language Scales (OWLS) for the training group as a function of speaking English as a second language (ESL) across the two time points**. Mean values of 12 non-ESL (open triangles) and 13 ESL students (filled triangles) are depicted. Vertical bars indicate standard errors of mean. Non-ESL participants tended to exhibit somewhat greater improvement in writing following training than ESL speakers. This difference, however, failed to reach statistical significance.

Reading and writing skills in the training group were systematically modulated by whether a student was a native English speaker or English was their second language (ESL). Although the mixed-model ANOVA on GMRT values resulted in a significant main effect of Time, with better scores at Time 2 (113.55 ± 2.42) than Time 1 (109.35 ± 2.40), *F*(1, 23) = 6.66, *p* < 0.017, $\eta_{p}^{2}=0.22$, this was subordinate to a two-way interaction with factor ESL, *F*(1, 23) = 7.72, *p* < 0.011, $\eta_{p}^{2}=0.25$ (see Figure 3). As can be seen in Table 2, native speakers of English improved significantly on the GMRT reading comprehension test across visits, *F*(1, 23) = 13.81, *p* < 0.002, and outperformed the trained ESL group after completion of the training, *F*(1, 23) = 4.96, *p* < 0.037. For the OWLS, significant main effects of ESL, *F*(1, 23) = 11.47, *p* < 0.003, $\eta_{p}^{2}=0.33$, and Time, *F*(1, 23) = 80.33, *p* < 0.001, $\eta_{p}^{2}=0.78$, were observed. Writing scores were higher overall in non-ESL than ESL speakers (105.04 ± 2.63 vs. 92.69 ± 2.53) and generally higher at Time 2, after FFW training than at Time 1, for both groups (111.39 ± 2.68 vs. 86.34 ± 1.83). These results show that the training led to significant improvement in writing for both ESL and non-ESL college students. The ESL × Time interaction failed to reach significance, *F*(1, 23) = 3.46, *p* < 0.076, $\eta_{P}^{2}=0.13$ As illustrated in Figure 4, while native speakers of English tended to exhibit somewhat greater enhancement in writing than the ESL group following FFW training, this difference did not reach significance.

**Table 2:** **Demographic characteristics and standardized literacy measures for the training group as a function of speaking English as a second language (ESL)**.

	ESL: no	ESL: yes
Sample size	*n* = 12	*n* = 13
Gender (male/female)	4/8	4/9
Age (years)	20.50 ± 4.76	19.69 ± 2.10
GMRT time 1	110.21 ± 10.70	108.49 ± 13.07
GMRT time 2	118.93 ± 13.96	108.17 ± 10.04
GMRT difference time 1 vs. 2	*p* < 0.002	*p* < 0.889
OWLS time 1	89.92 ± 6.69	82.77 ± 10.95
OWLS time 2	120.17 ± 15.28	102.62 ± 11.39
OWLS difference time 1 vs. 2	Not applicable	Not applicable

*Means ± standard deviations are shown; *p*-values are based on contrast analyses with a significance level set to 5%; due to a non-significant Time × ESL interaction, *post hoc* contrasts were not applicable for the OWLS. GMRT, Gates MacGinitie Reading Test; OWLS, oral and written language scales, written expression scale*.

## Discussion

### Summary of results

Overall this study provides evidence that both the reading and writing abilities of college students can be rapidly and substantially improved through the use of a series of neuroplasticity-based cognitive and linguistic training programs (FFW-L and Reading levels 3–5). College students who began the study with writing scores approaching a full standard deviation below the mean of average, based on a standardized, authentic writing assessment (OWLS, Written Expression Scale), who had been recalcitrant to traditional academic writing instruction approaches, showed significant improvement in writing after completing 11 weeks of daily training. These results show that from pre-training to post-training these students improved their writing abilities by one and two thirds standard deviations, moving from below to above average writing scores. A comparison group of college students with average writing scores, who did not receive training, showed no significant test-retest change in their writing scores over a comparable period of time. Results showed that at the onset of the study the writing scores of the training group participants were significantly lower than those of the comparison group. However, after the training this pattern of performance was reversed, with the trained group’s standard scores on the OWLS written expression scale now significantly exceeding those of the comparison group. The reading ability of these students was also assessed both before (pre-test) and after (post-test) the students with weak writing skills participated in the training program. It is of interest that the students with weak writing skills performed well within the normal range on reading at pre-test, albeit lower than the comparison group, on a standardized reading test (GMRT). At post-test the reading scores of the comparison group did not change while those of the group that received the training improved significantly.

Half of the students who entered the study with weak writing skills were native English language speakers while the others spoke ESL. These subgroups responded somewhat differently to the training. Both groups scored above average on the GMRT reading test before training. However, only the native English speakers showed significant gains in reading after training. The results for writing outcomes showed a different pattern. While both subgroups entered the study with substantially below average writing abilities as measured by the OWLS Written Expression Scales, the ESL student’s writing skills were lower than the native English speakers at pre-test. However, both the native and ESL speakers showed substantial benefits from the training, with both groups significantly improving their writing performance at post-test.

### Theoretical implications

The computerized training programs used in this study were developed based on a neurodevelopmental model that posits a continuum from perceptual/cognitive abilities, to spoken language abilities to written language abilities. Early perceptual and cognitive skills (attention, processing speed, sequencing, memory) are reported frequently in the research literature as concomitant individual differences in young children that correlate with and predict individual differences in language development (Heim and Benasich, 2006; Benasich and Choudhury, 2012). For example, Benasich and Tallal (2002) have shown that thresholds in the speed of auditory processing obtained in infancy are highly predictive of subsequent language expression and comprehension in preschool children. Similarly, it has been shown that spoken language development is highly predictive of early reading development and disorders (Flax et al., 2009). There also is a well-established relationship between individual differences in early reading and writing skills (Fitzgerald and Shanahan, 2000). Based on these relationships Tallal and colleagues posited a continuum between perceptual/cognitive abilities, particularly auditory processing speed, spoken language development, and written language development (Tallal, 2004). The Fast ForWord series of training exercises were developed specifically with this continuum in mind to help students struggling with language and literacy skills. The exercises are designed to go back to first principles of clarifying the neural representation of sounds within syllables, words and sentences as well as explicitly gaining mastery over all of the rules of English grammar. Training of these basic skills is presented within a context of a highly systematic and developmentally informed series of exercises that progressively challenge linguistic as well as processing and cognitive skills. This is done within the context of spoken language only (Fast ForWord-Literacy) until the student achieves a high level of mastery over the content across all exercises. Only then is the student introduced to exercises that include written material (Fast ForWord Reading Series). Like the spoken language exercises, the exercises in the reading series have been designed to follow a developmental trajectory ranging from phonemic awareness, to morphological awareness, to increasingly challenging aspects of reading comprehension within simple sentences to complex texts. Across all exercises, neuroplasticity-based learning principles are used to drive individually adaptive increases in performance, mouse-click-by-mouse-click. According to these learning principles, neuroplasticity is driven most efficiently by frequent and intense practice, sustained attention, individually adaptive trials (from easy to harder), and highly timed rewards and correction of errors (immediately following each response). Students progress at their own pace along a defined trajectory from easier items with lower cognitive load to items that are progressively more challenging both linguistically and cognitively. Students do not progress to harder more challenging items until they have shown a very high degree of mastery of easier items and levels.

When first examining the actual exercises included in the FFW-L and Reading training exercises, which are presented like repetitive video games, most teachers would likely consider them far too elementary to help secondary students, much less those who have been admitted to college, but are, nonetheless, struggling with writing. Intervention for students at this level generally focuses at a much higher level of content analysis and comprehension, organizational skills, and writing strategies for integrating newly learned materials into a cogent essay. It is assumed that by the time children move beyond elementary and middle school they have sufficient basic cognitive skills and have acquired proficiency in the basic linguistic skills and formal writing conventions they will need to handle the increasingly complex reading and writing demands placed on them in high school and college. However, based on standardized high stakes tests, this is not the case for an increasing majority (73% according to the 2012 NAEP results) of U.S. students. These numbers are even more discouraging for underrepresented minority and ESL students. The results of this study demonstrate the significant benefits of providing basic cognitive and linguistic skills remediation as an adjunct to more traditional methods for improving literacy skills in struggling students, well beyond the elementary school years.

### Comparison with previous studies

Not all studies that have used the Fast ForWord training approach have shown significant improvement in literacy. However, this study differed from previous studies in several ways. This is the first study to focus on college students. The study is also the first to focus on improving writing as well as reading. Furthermore, unlike previously published studies, this study used the full series of the Fast ForWord language and reading training programs, providing individually progressive training aimed at strengthening basic auditory processing and cognitive skills (memory, attention, processing speed, sequencing), spoken language skills (from phonology to syntax), reading skills (ranging from syllable, to word, to multiple paragraphs), to basic writing conventions (including spelling patterns and punctuation). Finally, it is important to emphasize that Fast ForWord differs from most other forms of computerized intervention in that it was not designed to be a stand alone software program. The intensity (5 days per week), fidelity to protocol, and student/monitor interaction required to achieve positive outcomes all require that the program be provided by an experienced Fast ForWord provider. Individual subject’s Fast ForWord performance on each exercise is analyzed daily *via* an electronic progress tracker. When used in clinics and classrooms, trained providers receive “red flags” that alert them to students who are experiencing problems on specific aspects of the training and have been trained how to intervene to correct these problems so that students can progress in the program. Studies that have not used a trained monitor who is experienced in providing this additional student support may have failed to get the full benefit of the intervention. This study benefited by having a highly trained and experienced Fast ForWord provider overseeing the daily implementation and student support.

### Limitations

There are several limitations to this study. This was not a randomized controlled trial. The study employed a quasi-experimental design in order to study the effects of cognitive, language, and reading training on the reading and writing skills of college students who were struggling with writing and compared them to a control group of average readers from the same university who did not receive training. The disadvantage of a quasi-experimental design is that groups are not equated on variables of interest at baseline and assigned randomly to receive the same treatment. As such, pre-specification of controls and other experimental variables are not able to be included to support strong statistical inferences. Subjects were recruited into the training group from two populations of students who historically are at risk for lower writing scores: students in developmental writing classes and underrepresented minority STEM majors. All students who volunteered to participate from these two groups were included in the training group. There was no attempt to include or exclude students who had a previous or current diagnosis of dyslexia or other learning disabilities. The comparison group received the same pre-tests and post-tests at the beginning and end of the study, but did not participate in any training or come to the lab daily during the semester. While this comparison group provided control for any changes that might occur from taking the reading and writing test more than once, as well as changes that might occur over a college semester, the effect of differential contact with the research staff across the course of the study cannot be assessed. The training group came to the study lab and completed 50 min of training 5 days a week for a full semester (11 weeks). There was no attempt to evaluate different durations of intervention. Furthermore, the experimental group received only one form of training in this study. Previous studies with younger students have demonstrated that it may be the intensity of training, rather than the specificity of the type of training that is most important for driving improvements in language and literacy (Gillam et al., 2008). It is not possible, therefore, to determine that the significant improvements in reading and writing found in this study are specific to the Fast ForWord program, specific to students struggling with writing, or the extent to which they may have been achieved by other training programs that were equally intensive. It is also not possible to determine the extent to which these results may be affected by the age of the students. Finally, a limited number of standardized tests were used as outcome measures. While these are well standardized tests that sample a broad range of authentic reading and writing skills, it will be important to replicate these results with other measures, particularly those assessing classroom performance. Further research will be needed to address these important variables. Specifically, it will be important to replicate these results using a randomized controlled study design as well as to determine the extent to which they may apply to younger students or be achieved by other methods. Finally, significant improvements in reading and writing were found immediately following training. It will be important to do follow-up testing over time to determine the longer-term effects of supplementing traditional college instruction with computer-based interventions for improving reading and writing outcomes in struggling students.

## Conclusion

By the time students reach college it is assumed, often incorrectly, that they do not need instruction or practice in basic language, reading, and writing skills. Rather, they are bombarded with increasingly complex lectures, reading, and writing assignments in virtually all of their courses. At the same time it is not unusual to hear college professors bemoan the fact that many of their students are unable to string two complex sentences together correctly, much less read and analyze complex material and write cogent papers synthesizing new knowledge and expressing their own thoughts and ideas. While many universities are offering developmental writing courses, these rarely focus on taking struggling students back to the basics and progressing systematically to higher levels.

The results of this study demonstrated that a neuroplasticity-based, computer training program, designed initially for younger struggling students to improve basic cognitive, language, and reading skills (Fast ForWord), could successfully be implemented in a college setting to help college students with below average writing abilities rapidly achieve above average writing skills. The results of this study support the efficacy of systematic, progressive perceptual/cognitive, language, and reading skills training for struggling students beyond the primary and secondary school level, as shown here in a college sample. This study also validates the positive benefits of using computer intervention strategies in college students that provide them with a concise, controlled, and individually adaptive means of significantly improving their basic language and literacy skills in a manageable amount of time, without unduly interfering with their intense program of college classes.

It is important to note that no explicit practice with writing *per se* is included in the training programs used in this study. Thus, the results of this study demonstrated that training in basic cognitive, listening, and reading skills generalize to improved writing ability. Our research design did not allow us to distinguish which of the many skills included in the training led to these improvements in writing. Future research is needed to determine the extent to which specific cognitive, language, or reading skills included within the series of training programs used in this study had the most impact on writing.

## Conflict of Interest Statement

Paula Tallal, Ph.D. is a Co-Founder and Director of Scientific Learning Corporation, the company that produces the Fast ForWord^®^ training programs used in this research.

## Supplementary Material

The Supplementary Material for this article can be found online at: http://www.frontiersin.org/Educational_Psychology/10.3389/fpsyg.2013.00137/abstract
